# Translucency of Dental Ceramic, Post and Bracket

**DOI:** 10.3390/ma8115379

**Published:** 2015-10-28

**Authors:** Yong-Keun Lee

**Affiliations:** Institute for Clinical Performance of Biomaterials (ICPB) and ETN Dental Clinic, 106-B101, 27 Heukseokhangang-ro, Dongjak-gu, Seoul 06981, Korea; ykleedm@gmail.com; Tel.: +82-2-816-1616; Fax: +82-2-816-0606

**Keywords:** translucency, dental ceramic, esthetic post, orthodontic bracket

## Abstract

Translucency of dental ceramics, esthetic posts and orthodontic brackets was reviewed. Translucency parameter (*TP*) and contrast ratio (*CR*) are generally used for translucency evaluation. For the evaluation of translucency, two criteria such as the translucency of human teeth (*TP* = 15–19, 1 mm thick) and the visual perceptibility threshold for the translucency difference (∆*CR* > 0.07 or ∆*TP* > 2) were used. In ceramics, translucency differences were in the perceptible range depending on the type of material and the thickness. However, variations caused by the difference in the required thickness for each layer by the material and also by the measurement protocols should be considered. As to the translucency of esthetic posts, a significant difference was found among the post systems. Translucency was influenced by the bracket composition and brand, and the differences by the brand were visually perceptible.

## 1. Introduction

Since human tooth enamel is highly translucent, translucency is a desirable characteristic of dental ceramic materials [1]. Translucency is emphasized as one of the primary factors in controlling the esthetic outcome because it makes restorations appear more natural [2,3]. Requirements for dental esthetic materials are contradictory since translucency of restorative materials influences the masking ability and color blending effect [4]. In addition, translucency of dental ceramics is closely related to the light transmission and light-curing efficiency of underlying resin-based luting agents [5].

As dental ceramics evolve and patients’ demands for esthetic restorations increase, guidance in selecting an appropriate ceramic system when faced with different esthetic demands was offered. Considering the translucency and the mechanical strength of dental ceramics simultaneously, high-translucency ceramics should be used when high-level esthetics are required, since high-strength ceramics tend to be more opaque and pose a challenge when trying to match tooth color [6]. All-ceramic restorations have been advocated for superior esthetics [7]. As to the optimization of the translucency of dental ceramics, the biomimetic simulation of natural tooth microstructure might be a promising method [4].

In the evaluation of optical properties, the Commission Internationale de l’Eclairage (CIE) LAB system is generally used [8]. The CIE L* coordinate ranges from 0 to 100 and represents the luminosity, the a* coordinate ranges from −90 to 70 and represents greenness (positive a*) and redness (negative a*), and the b* coordinate ranges from –80 to 100 and represents yellowness (positive b*) and blueness (negative b*), and the CIE standard illuminants are generally used [8]. Chroma is calculated as C*_ab_ = (a*^2^ + b*^2^)^1/2^, and the color difference is calculated as ∆E*_ab_ = (∆L*^2^ + ∆a*^2^ + ∆b*^2^)^1/2^. The advantage of this system over the Munsell color system composed of hue, chroma and value attributes is that the CIELAB coordinates are evenly spaced in terms of visual perception, so that the spectral readings can be correlated with subjective observations [9,10].

Translucency is the relative amount of light transmission or diffuse reflection from a substrate surface through a turbid medium [4,11]. While the term translucency is used to describe the optical property, the corresponding term transmission is a physical term which represents the ability of a medium to permit light to pass through it [4,12]. The Lambert-Beer law relates the attenuation of light to the properties of the material through which the light is traveling. The absorption coefficient (*α*) and the constant value related to the reflection (C) of the samples are obtained from the relationship of the natural logarithm (*ln*) of the transmission coefficient (*T*) to the specimen’s thickness. The general equation of the Lambert-Beer law is given by *T* = C*e−^αd^*, where *T* is the transmission coefficient, C the constant related to the reflection material, *e* the Naperian number, *α* the coefficient of absorption, and *d* the sample thickness [13,14]. Translucency can be defined as the ratio of the intensity of the transmitted light to that of the incident light. Opacity is the inverse of the translucency, and optical density is the common logarithm of the opacity. In the development of a method for measuring translucency, the following facts proved to be important: (1) only visible light is of interest; (2) the eye is most sensitive to a wavelength of approximately 550 nm, and (3) light transmitted through a body decreases exponentially with an increase in thickness [1]. Translucency can be studied by measuring direct transmission (when light goes through without a change in direction or quality), total transmission (combination of direct and diffuse transmission) and spectral reflectance (fraction of incident light that is reflected at an interface such as porosity) [15].

Regarding that the measurement of translucency, translucency parameter (*TP*), contrast ratio (*CR*) [7,16,17], and direct and total transmission (τ, %) [11,18] are generally determined, and the correlations among these values were confirmed [4]. The *TP* value is the color difference (∆E*_ab_) of the same specimen over ideal white and black backgrounds [19], and the range of the *TP* could be 0 to 100. The *CR* value is calculated from the spectral reflectance of the specimens with black (*Y_b_*) and white (*Y_w_*) backgrounds to give *Y_b_*/*Y_w_* [20]. In the *CR*, the value ranges from 0 to 1, with 0 as totally transparent and 1 as totally opaque. The correlation between the *TP* and the *CR* values was assessed based on dental ceramics [21]. Specimens of 0.7 mm in thickness were fabricated (This content was from reference [21].) for the following materials: Vita VM9 (Vita Zahnfabrik, Bad Säckingen, Germany, positive control), Vita PM9 (Vita Zahnfabrik), IPS Empress CAD (Ivoclar Vivadent, Schaan, Liechtenstein), IPS e.max CAD (Ivoclar Vivadent), IPS e.maxPress (Ivoclar Vivadent) and Lava Zirconia (3M ESPE, St. Paul, MN, USA, negative control). In the results, a significant correlation was found (*r* = −0.99).

A wide range of reported translucency values indicates the need for the establishment of criteria for the interpretation of the values [22]. Therefore, two criteria of the translucency of human teeth (*TP* = 15–19) and the visual perceptibility threshold for the translucency difference (∆*CR* > 0.07 or ∆*TP* > 2) were applied in this review. The *TP* values from 15 to 19 were reported based on 1-mm-thick human teeth [2,23], which varied by the measurement protocols and also by the region of tooth. The relationship between instrumental translucency values and subjective visual assessment of differences in dental ceramic translucency was determined [24], and the overall mean visual perceptibility threshold in the *CR* difference (∆*CR*) was 0.07. To apply this threshold to the *TP* value, it was transformed into a ∆*TP* value of 2 using the regression equation between the *TP* and the *CR* values previously reported [25]. As to the categorization of translucency in dental resin composites, the values were categorized into three groups at the *TP* values of 13.0 and 18.0 [25], or were divided into three equal segments [22].

The objective was to review the translucency of dental ceramics, esthetic posts and orthodontic brackets based on the peer-reviewed articles and related references. The two criteria of the translucency of human teeth and the visual perceptibility threshold for the translucency difference were applied.

## 2. Translucency of Dental Ceramics

### 2.1 Range of Translucency in Ceramics

There are substantial variations among the reported translucency values for dental ceramics [24]. Translucency of comparable shades of five porcelains was determined [11]. Both direct and total transmission were measured based on 1-mm-thick specimens. In the results, the values for direct transmission were averaged 0.13%, whereas the values for total transmission were averaged 26.8%, which varied by the brand and shade. Spectral transmission of porcelain laminate veneers was measured at three thicknesses (0.5, 0.75 and 1 mm) and three opacity groups (25%, 75% and 100%) [18]. The results indicated that the thickness of the veneer was the primary factor affecting light transmission and not the opacity group. Although the marked opacifying effect that porosity has on dental porcelain was reported [1,5], experimental results [26] were different. The effects of the powder (*P*)/liquid (*L*) ratio during condensation on the porosity (%) and the *CR* values of four dental porcelains were determined [26]. However, the total porosity of specimens was found to be sensitive to the *P*/*L* ratio, whereas the *P*/*L* ratio did not significantly affect the *CR* value.

It was reported that the *CR* value of Empress 2 (Ivoclar Vivadent) ranged from 0.19 to 0.27 at 0.8-mm-thick specimens [27]. The *CR* values of six all-ceramic core materials at clinical thicknesses were compared with dentin porcelain [7]. Specimens of 0.5 mm in thickness were fabricated with heat-pressed (IPS Empress dentin, IPS Empress 2 dentin), slip-cast (In-Ceram Alumina, In-Ceram Spinell, In-Ceram Zirconia manufactured by Vita Zahnfabrik), and sintered (Procera AllCeram by Nobel Biocare, Göteborg, Sweden). In the results, the ranges were dentin porcelain (*CR* = 0.60) > In-Ceram Spinell (0.67) > Empress (0.72), Procera (0.72), Empress 2 (0.74) > In-Ceram Alumina (0.87) > In-Ceram Zirconia (1.0). The *CR* values of the six all-ceramic cores with their corresponding veneer porcelains were compared with core-only specimens [16]. The *CR* values of six all-ceramic materials [7] veneered at clinically appropriate thicknesses (final thickness: 1.5 mm) were measured and the increased *CR* values ranging from 0.78 to 1.0 were reported. Measurements were repeated after a glazing cycle, and the glazing cycle resulted in increased translucency for all the materials except for the completely opaque materials. The *CR* values of four core ceramics were assessed based on 0.5-mm-thick specimens [17]. In the results, the translucency of Empress 2 dentin core (0.78) was significantly higher than In-Ceram Alumina (0.94), In-Ceram Zirconia (1.0) and Cercon (Dentsply Ceramco, Burlington, VT, USA, 1.0). Therefore, translucency differences of core and veneered ceramics were in the perceptible range (∆*CR* > 0.07) [24,25].

With dental porcelain and porcelain-repairing resin composites, the *TP* values relative to the CIE standard illuminant D65 (daylight condition), A (incandescent lamp) and F2 (fluorescent lamp) were compared [28]. In the results, the *TP* values were influenced by the illuminant, and the translucency under the illuminant D65 was lower than that under the A or F2. Differences in the *TP* of all-ceramic core, veneer and layered specimens relative to the illuminants D65, A and F2 were also determined [29]. In the results, differences in the *TP* values by the illuminant (∆*TP*) were influenced by the three factors of type of the ceramic, the brand/thickness of the ceramic and the illuminant. Higher translucent materials showed higher ∆*TP* values, and the *TP* values relative to the illuminants A and F2 were higher than those relative to the D65.

### 2.2 Influence of Microstructure

Translucency of dental ceramics is generally influenced by factors such as crystalline structure, grain size and pigments, as well as number, size and distribution of defects and porosity [5]. If the crystals are smaller than the wavelength of visible light, the ceramic would appear transparent; however, in the case of light scattering and diffuse reflection by bigger crystals, the ceramic would appear opaque. Scattering within the body diminishes the emergent beam intensity most effectively [30]. Within the body of the ceramic, a portion of the light may be absorbed [1]. Reflections and refractions of light at the interfaces between phases scatter the light. Therefore, the degree of scattering at the interfaces between adjacent crystals and between crystals and the glass phase is a function of the relative refractive indices of the different phases, and their particle sizes, shapes and volume concentrations [1,31]. The translucency of glass-ceramics is dependent on the heat treatment temperature that induces crystal nucleation and growth process [32].

It is difficult to prepare highly translucent ceramic-glass composites for dental restorations due to the light scattering that occurs in multiphase ceramics. The effectiveness of a systematic approach in designing specific glass compositions to prepare high translucent glass-infiltrated ceramic composites was verified [33]. First, it was necessary to calculate the viscosity of glass at the infiltration temperature. Then, a glass composition was designed for targeted viscosity and refractive index. In the results, the light transmission of the prepared composite was significantly higher than a commercial ceramic-glass composite, due to the matching of glass and refractive indices which decreased the scattering and absorption.

Manufacturers offer high translucent (HT) and low translucent (LT) versions of CAD (computer aided design)/CAM (computer aided manufacturing) blocks. A small number of large crystals are present in HT material, whereas LT material contains a large number of smaller crystals [34]. In the last decade, zirconia has generated interest due to its favorable mechanical and biological properties. However, zirconia lacks the translucency and therefore has limitations, especially in esthetic cases [15]. The light transmission of zirconia was compared with lithium disilicate [15]. Specimens of LT and HT versions of zirconia (Metoxit Pre-Sintered Zirconia by High Tech Ceramics, Thayngen, Switzerland) and lithium disilicate (IPS e.max) were fabricated. Diffuse light transmission was measured, followed by a microstructural analysis. In the results, HT lithium disilicate showed the highest transmission, followed by LT lithium disilicate, HT zirconia and LT zirconia. The light transmission of the materials correlated to their microstructure. Despite manufacturers’ efforts to make zirconia more translucent, the light transmission still did not match lithium disilicate. Therefore, more research is required towards making zirconia more translucent for its potential use in esthetic monolithic restoration. The translucency of Y-TZP (yttria-stabilized tetragonal zirconia polycrystal) all-ceramic (IPS e.max ZirCAD block by Ivoclar Vivadent) restorations by different veneering techniques was compared [35]. The 0.5-mm-thick specimens were divided into three groups. Group 1 was veneered by the fully anatomical technique, group 2 was veneered by the traditional layering technique and group 3 was veneered by the cutback technique. In the results, the group 1 was the most translucent and lightest while the group 3 was the least translucent and the darkest. The total luminous transmission (τ) was in the range of 1.5 to 1.6 after veneering, while it was 2.3 before veneering.

The intensity of incident light is reduced in several ways between incidence and final emergence. Since some reflections occur at the entry face, this surface should be standardized [1]. Absolute translucency of monolithic CAD/CAM materials and direct resin composites was evaluated with respect to thickness and surface roughness [36]. Specimens of three CAD/CAM glass ceramics (CELTRA Duo by Dentsply DeguDent, Hanau-Wolfgang, Germany, IPS e.max CAD, IPS Empress CAD), feldspathic ceramic (Vita Mark II by Vita Zahnfabrik), hybrid ceramic (Vita Enamic by Vita Zahnfabrik), resin nanoceramic composite (Lava Ultimate by 3M ESPE), experimental CAD/CAM nanohybrid resin composite, two interim materials and three resin composites were fabricated. After three surface treatments (SiC P2000, P1200 or P500), total light transmission and surface roughness values were measured. In the results, the greatest influence on the transmission was thickness, followed by material and the treatment method. Transmission values seemed to be material-specific; therefore, no generalization relating to the different material classes could be made. All CAD/CAM ceramics demonstrated a greater decrease in translucency after rough polishing than did the resin-based materials, indicating sufficient polishing of the restoration essential. The effect of polishing on the surface gloss of monolithic zirconia was estimated and the *TP* values were compared [37]. Four monolithic partially stabilized zirconia brands [Prettau (PRT) by Zirkonzahn, Taufers, Italy, Bruxzir (BRX) by Glidewell, Irvine, CA, USA, Zenostar (ZEN) by Ivoclar Vivadent, and Katana (KAT) by Kuraray Noritake, Tokyo, Japan] and one fully stabilized zirconia [Prettau Anterior (PRTA)] were used to fabricate specimens of 0.5, 0.7, 1.0, 1.2, 1.5 and 2.0 mm. Zirconia core material [ICE Zircon (ICE) by Zirkonzahn] was used as a control. In the results, surface gloss was significantly affected by polishing regardless of brand and thickness. The *TP* values ranged from 5.7 to 20.4 before polishing and from 5.1 to 20.0 after polishing. The ranking from least to highest translucent (after polish) was: BRX = ICE = PRT < ZEN < KAT < PRTA. Therefore, they showed perceptible translucency differences (*∆TP* > 2) [24,25] between the materials, and several materials showed similar translucency to that of human teeth (*TP* = 15–19) [2,23].

### 2.3. Influence of Ceramic Translucency on Light-Curing of Luting Materials

Translucency of dental ceramics influences the degree of light curing of light-cured or dual-cured materials underlying the ceramics. Light passing through dental ceramics, such as laminate veneers and ceramic brackets, is important for adhesive luting, since many dual-cured luting materials are sensitive to additional light-curing [38]. The degree of monomer conversion (DC) in varied resin cement shades was evaluated when light-cured through varied feldspathic ceramic shades [39]. The light transmission of 2-mm-thick specimens was calculated. To measure the DC, resin cement specimens (100 μm) were light-cured under a ceramic block (2 mm thick). In the results, the transmission of ceramics, by the shade, was 12.4% (0M1 shade), 5.8% (2M2) and 1.1% (5M3). The DC of resin cement shades cured under 5M3 shade was significantly lower than the other groups. The amounts of light passing (irradiance) through five shaded zirconia were determined with respect to material thickness (0.5, 1, 1.5, 2, 2.5 and 3 mm), exposure distance and different curing modes [38]. Irradiance of curing light was measured at varying exposure distances, ranging from direct contact to a distance of 7 mm increasing in 1 mm steps. The results indicated that the use of dual-cured cements with less light requirement was recommended for restorations thicker than 1.5 mm in light-shaded zirconia and 0.5 mm in darker-shaded zirconia.

The effect of monolithic zirconia thickness on irradiance and total irradiant energy transmitted through each specimen was quantified [37]. In the results, the ranking from least to highest total irradiant energy was: Bruxzir < Prettau < ICE Zircon = Zenostar < Katana = Prettau Anterior. There was an inverse relationship between translucency, irradiant energy and thickness of zirconia. Therefore, brand selection, thickness and polishing of monolithic zirconia could affect the ultimate clinical outcome of the optical properties of zirconia restorations. The effects of ceramic veneer thicknesses on the light-curing of resin cements were evaluated [40]. Specimens were fabricated by using the LT version of pressable ceramic (IPS e.maxPress). Specimens were divided into light-cured and dual-cured and each group was further divided into four subgroups, based on ceramic thickness (0.3, 0.6, 0.9 and 1.2 mm). In the results, the degree of conversion and hardness of the resin cement was unaffected by veneer thicknesses between 0.3 and 0.9 mm. However, the dual-cured cement group resulted in a significantly lower DC for the 1.2 mm subgroup. While clinically adequate curing of light-cured cement could be achieved with a maximum 1.2 mm of veneer restoration, the increase of curing time or light intensity was clinically needed for the dual-cured resin cements at the thickness of more than 0.9 mm.

Since the amount of passed light through ceramic-based restorations and orthodontic brackets influences the performance of light-cured or dual-cured resin-based luting materials, the translucency of overlaying ceramics should be carefully considered when used in clinical situations. The opacity and thickness of overlaying ceramics influence the light transmission.

## 3. Range of Translucency in Esthetic Post and Bracket

Cast-metal post-core systems have a long history of successful use. However, their high elastic modulus can cause root fractures [41,42]. Increasing demand for more esthetically appealing and biocompatible restorations has led to the development of tooth-colored, translucent, metal-free post systems [43]. It was verified whether the light transmitting ability of marketed fiber posts reflected the manufacturers’ claims for translucency [44]. The amount of photons reaching different post levels was measured. In the results, light intensity decreased from coronal to apical and rose again at the apical tip. A significant difference in translucency was found among the post systems. The DC of dual-cured resin cement was evaluated when used to lute fiber posts with different translucencies [45]. For Light-Post (Bisco, Schaumburg, IL, USA), resin cement at deep depth showed the lowest DC and no significant difference in DC was found between the other depths. For Aestheti-Post (Bisco), superficial depth presented higher DC values than those in the medium and deep depths. The effect of fiber post translucency on the push-out bond strength to dentin was evaluated [45]. Bovine roots were endodontically treated, and Light-Post and Aestheti-Post were cemented. The roots/cemented posts were transversally sectioned and the push-out test was performed. In the results, there was no difference between the two fiber posts. Zirconia ceramic post systems have been introduced to satisfy the heightened awareness of esthetics, whereby the translucency of all-ceramic crowns can be successfully maintained with the use of ceramic post-core materials [46]. Light transmission and esthetic advantages of zirconia posts were evaluated [47]. In the results, zirconia posts were shown to improve the esthetic quality of all-ceramic crowns. With the zirconia material, its main advantages lie in its translucency and tooth-colored shade, thereby rendering the material usable with all-ceramic crowns in the anterior region.

Variations in optical properties of esthetic brackets influenced the esthetic performance and the DC of the adhesives through the brackets [48]. As to the translucency of esthetic brackets, direct light transmission in varied types of brackets was investigated, and the results were correlated with their structure, morphologic factors and composition [49]. Eight brackets were investigated, and spectroscopic direct transmission analysis was performed at 320 to 700 nm. From each spectrum, the amount of light transmission corresponding to the peak absorbance wavelength of the photoinitiator (468 nm) was recorded. In the results, the highest light transmission value was obtained from monocrystal bracket (Starfire by A Company, San Diego, CA, USA: 35.0%), followed by polycrystalline (Fascination by Dentaurum, Pforzheim, Germany: 5.7%) and ceramic/polycarbonate base bracket (Ceramaflex by TP Orthodontics, La Porte, IN, USA: 4.0%). The results showed that the structure of the brackets by design, morphologic factors of each bracket, and composition of the brackets, such as polymer, momo- or polycrystalline ceramic, were found to affect direct light transmission significantly. Diffuse light transmission of esthetic brackets was determined [48]. Four ceramic and four plastic brands were evaluated. In the results, diffuse light transmission was influenced by bracket brand and ranged from 44.9% to 75.9%. Translucency of esthetic brackets also was determined using a spectroradiometer [50]. The *TP* and the *CR* values at central and tie wing regions of four plastic and four ceramic brackets were determined. In the results, the *TP* and the *CR* values for the central region were 16.4–27.7 and 0.38–0.58, and those for the tie wings were 24.0–39.9 and 0.25–0.45, respectively. The *TP* and the *CR* values were significantly influenced by the bracket composition and brand, and differences in the translucency of brackets by brand were in the visually perceptible range (∆*CR* > 0.07) [24]. The bond strength differences afforded by the type of curing light were compared [51]. In the bonding of brackets to human teeth, the plasma arc curing light and the LED (light emitting diode) curing light were used to cure the resin composite for four, six and eight seconds. The results showed that the LED curing light produced bond strength sufficient for maintaining the bracket even with a short burst of curing. Therefore, it was expected that the LED curing light would be readily accepted by orthodontists.

Optical properties of esthetic brackets were evaluated and their influence on visual perception was determined [52]. The translucency of 16 commercial brands of esthetic brackets was measured. In the results, the direct transmission of light ranged from 0.0% to 38.8% and the optical properties of esthetic brackets have a direct influence on visual perception. Esthetic color performance (color blending) of plastic and ceramic brackets was evaluated by determining the color changes of shade guide tabs before and after bracket placement [53]. Brackets were placed on the labial surface of the A1 and A4 tabs of a Vitapan classic shade guide. In the results, all the investigated esthetic brackets induced clinically unacceptable (∆E*_ab_ > 5.5) color changes when placed on the shade tabs. Based on a spectroradiometer measurement, a mean color difference of 2.6 ∆E*_ab_ units is considered to be clinically perceptible, while a mean color difference of 5.5 ∆E*_ab_ units is considered to be clinically unacceptable [54]. Esthetic color performance of brackets on the less chromatic and lighter tab was better than that on the more chromatic and dark tab. High translucency of the bracket alone did not lead to better aesthetic color performance.

## 4. Conclusions

In dental ceramics, translucency differences were in the perceptible range depending on the type of materials and thickness (∆*CR* > 0.07 or ∆*TP* > 2). However, variations in translucency indices caused by the difference in the required thickness for each layer by the material and also by the measurement protocols should be considered for comparison. As to the esthetic posts, a significant difference in translucency was found among the post systems. The translucency of esthetic brackets was significantly influenced by the bracket composition and brand, and differences in the translucency of brackets by brand were visually perceptible (∆*CR* > 0.07). These variations in translucency should be considered in the selection of ceramic-based materials. Also, differential requirements for each material should be clearly set up and confirmed. In the future, varied translucency indices should be unified to a single index to facilitate the comparison and evaluation of the translucency values.
